# Impact of drainage retinotomy on surgical outcomes of retinal detachment: insights from the Japan-Retinal Detachment Registry

**DOI:** 10.1038/s41598-024-58453-5

**Published:** 2024-04-02

**Authors:** Hisashi Fukuyama, Hiroto Ishikawa, Fumi Gomi, Japan-Retinal Detachment Registry Group, Shuichi Yamamoto, Takayuki Baba, Eiju Sato, Masayasu Kitahashi, Tomoaki Tatsumi, Gen Miura, Tomohiro Niizawa, Taiji Sakamoto, Keita Yamakiri, Toshifumi Yamashita, Hiroki Otsuka, Seiji Sameshima, Narimasa Yoshinaga, Shozo Sonoda, Akito Hirakata, Takashi Koto, Makoto Inoue, Kazunari Hirota, Yuji Itoh, Tadashi Orihara, Yoshinobu Emoto, Masahiko Sano, Hiroyuki Takahashi, Ryo Tokizawa, Hidetoshi Yamashita, Koichi Nishitsuka, Yutaka Kaneko, Katsuhiro Nishi, Akitoshi Yoshida, Shinji Ono, Hiroyuki Hirokawa, Kenji Sogawa, Tsuneaki Omae, Akihiro Ishibazawa, Shoji Kishi, Hideo Akiyama, Hidetaka Matsu-moto, Ryo Mukai, Masahiro Morimoto, Mitsuru Nakazawa, Yukihiko Suzuki, Takashi Kudo, Kobu Adachi, Susumu Ishida, Kousuke Noda, Satoru Kase, Syouhei Mori, Ryo Ando, Michiyuki Saito, Tomohiro Suzuki, Kanji Takahashi, Yoshimi Nagai, Tadashi Nakauchi, Haruiko Yamada, Shuji Kusaka, Daishi Tsujioka, Akitaka Tsujikawa, Kiyoshi Suzuma, Tatsuro Ishibashi, Koh-Hei Sonoda, Yasuhiro Ikeda, Riichiro Kohno, Keijiro Ishikawa, Mineo Kondo, Maki Kozawa, Takashi Kitaoka, Eiko Tsuiki, Yuichiro Ogura, Munenori Yoshida, Hiroshi Morita, Aki Kato, Yoshio Hirano, Kazuhiko Sugitani, Hiroko Terasaki, Takeshi Iwase, Yasuki Ito, Shinji Ueno, Hiroki Kaneko, Norie Nonobe, Taro Kominami, Noriyuki Azuma, Tadashi Yokoi, Hiroyuki Shimada, Hiroyuki Nakashizuka, Takayuki Hattori, Ari Shinojima, Yorihisa Kutagawa, Fumio Shiraga, Yuki Morizane, Shuhei Kimura, Tsunehiko Ikeda, Teruyo Kida, Takaki Sato, Masanori Fukumoto, Kazuyuki Emi, Hiroshi Nakashima, Masahito Ohji, Masashi Kakinoki, Osamu Sawada, Shinobu Takeuchi, Sumiyoshi Tanaka, Tomohiro Iida, Hideki Koizumi, Ichiro Maruko, Taiji Hasegawa, Akiko Kogure, Hiroyuki Iijima, Tomohiro Oshiro, Yasushi Tateno, Wataru Kikushima, Atsushi Sugiyama, Seigo Yoneyama, Kazuaki Kadonosono, Shimpei Sato, Shin Yamane

**Affiliations:** 1 Department of Ophthalmology, Hyogo Medical University, 1-1 Mukogawa-Cho, Nishinomiya, 663-8501 Japan; 2 Department of Ophthalmology, Mirai Eye & Skin Clinic, Osaka, Japan; 3 Chiba University, Chiba, Japan; 4 Kagoshima University, Kagoshima, Japan; 5 Kyorin University, Tokyo, Japan; 6 Yamagata University, Yamagata, Japan; 7 Asahikawa Medical University Hospital, Asahikawa, Japan; 8 Gunma University, Gunma, Japan; 9 Hirosaki University, Hirosaki, Japan; 10 Hokkaido University, Sapporo, Japan; 11 Kansai Medical University Hospital, Hirakata, Japan; 12 Kindai University Sakai Hospital, Sakai, Japan; 13 Kyoto University, Kyoto, Japan; 14 Kyushu University, Fukuoka, Japan; 15 Mie University, Tsu, Japan; 16 Nagasaki University, Nagasaki, Japan; 17 Nagoya City University, Nagoya, Japan; 18 Nagoya University, Nagoya, Japan; 19 National Center for Child Health and Development, Tokyo, Japan; 20 Nihon University Hospital, Tokyo, Japan; 21 Okayama University, Okayama, Japan; 22 Osaka Medical School, Osaka, Japan; 23 Osaka Rosai Hospital, Osaka, Japan; 24 Shiga Medical University, Otsu, Japan; 25 Takeuchi Eye Clinic, Tokyo, Japan; 26 Tokyo Womens Medical College, Tokyo, Japan; 27 Yamanashi University, Kofu, Japan; 28 Yokohama City University Medical Center, Yokohama, Japan

**Keywords:** Medical research, Outcomes research, Risk factors

## Abstract

We investigated the impact of drainage retinotomy on the outcome of pars plana vitrectomy for repair of rhegmatogenous retinal detachment (RRD). This study was a retrospective observational multicenter study. All patients were registered with the Japan-Retinal Detachment Registry. We analyzed 1887 eyes with RRD that had undergone vitrectomy and were observed for 6 months between February 2016 and March 2017. We compared the baseline characteristics and postoperative outcomes between eyes with and without drainage retinectomy. We then performed propensity score matching using preoperative findings as covariates to adjust for relevant confounders. Of 3446 eyes, 1887 met the inclusion criteria. Among them, 559 eyes underwent vitrectomy with drainage retinotomy, and 1328 eyes underwent vitrectomy without drainage retinotomy. After propensity score matching, each group comprised 544 eyes. There was no significant difference between the two groups in BCVA at 6 months after vitrectomy (0.181 vs. 0.166, P = 0.23), the primary anatomical success rate (6.3% vs. 4.4%, P = 0.22), or the rate of secondary surgery for ERM within 6 months (1.5% vs. 1.3%, P = 1.0). Drainage retinectomy does not increase the risk of decreased postoperative BCVA, surgical failure, or secondary surgery for ERM within six months outcomes.

## Introduction

Rhegmatogenous retinal detachment (RRD) is a common cause of visual impairment and a serious ocular condition that necessitates a prompt surgical intervention to prevent blindness. RRD is due to retinal breaks with accumulation of fluid between the neurosensory retina and the retinal pigment epithelium. Recent progress in pars plana vitrectomy (PPV), such as advances in vitrectomy instrumentation, visualization systems, and surgical techniques, have led to improvements in surgical outcomes^1^. PPV is an effective treatment for RRD in terms of providing anatomical success^2–4^.

Drainage retinotomy is a surgical technique that has played an effective role in PPV for RRD. This technique involves the creation of a small retinal hole to assist in the removal of subretinal fluid for subsequent reattachment and laser coagulation of the retinal tear^5^. Drainage retinotomy is important in the management of RRD because it effectively removes subretinal fluid, thus contributing to successful retinal reattachment and avoiding complications such as retinal folds^6^.

A recent retrospective study showed that drainage retinotomy is a significant risk factor for surgical failure in patients undergoing PPV for primary RRD^7^. By contrast, other retrospective studies have shown no influence of drainage retinotomy on the primary anatomical success rate^8–10^. The impact of drainage retinotomy on the outcome of PPV performed for repair of RRD remains unclear. In addition, the above-mentioned retrospective studies were conducted in a single center. The potential bias in surgical techniques, experiences, and patient populations among a limited group can influence the results. A multicenter approach, including a wider range of patient demographics and surgeons, provides a more accurate assessment of the impact of drainage retinotomy on patient outcomes.

The Japan-Retinal Detachment (J-RD) Registry collected data from 26 institutions across Japan^11^. The J-RD Registry has yielded several noteworthy findings as demonstrated by a series of published studies^12–15^. Therefore, we investigated the impact of drainage retinotomy on the short term outcome PPV performed for repair of RRD using the J-RD Registry. We anticipated that the findings of this study would provide valuable insights into the risks and benefits of drainage retinotomy, informing clinical decision-making and potentially improving patient outcomes.

## Results

**Figure 1:**
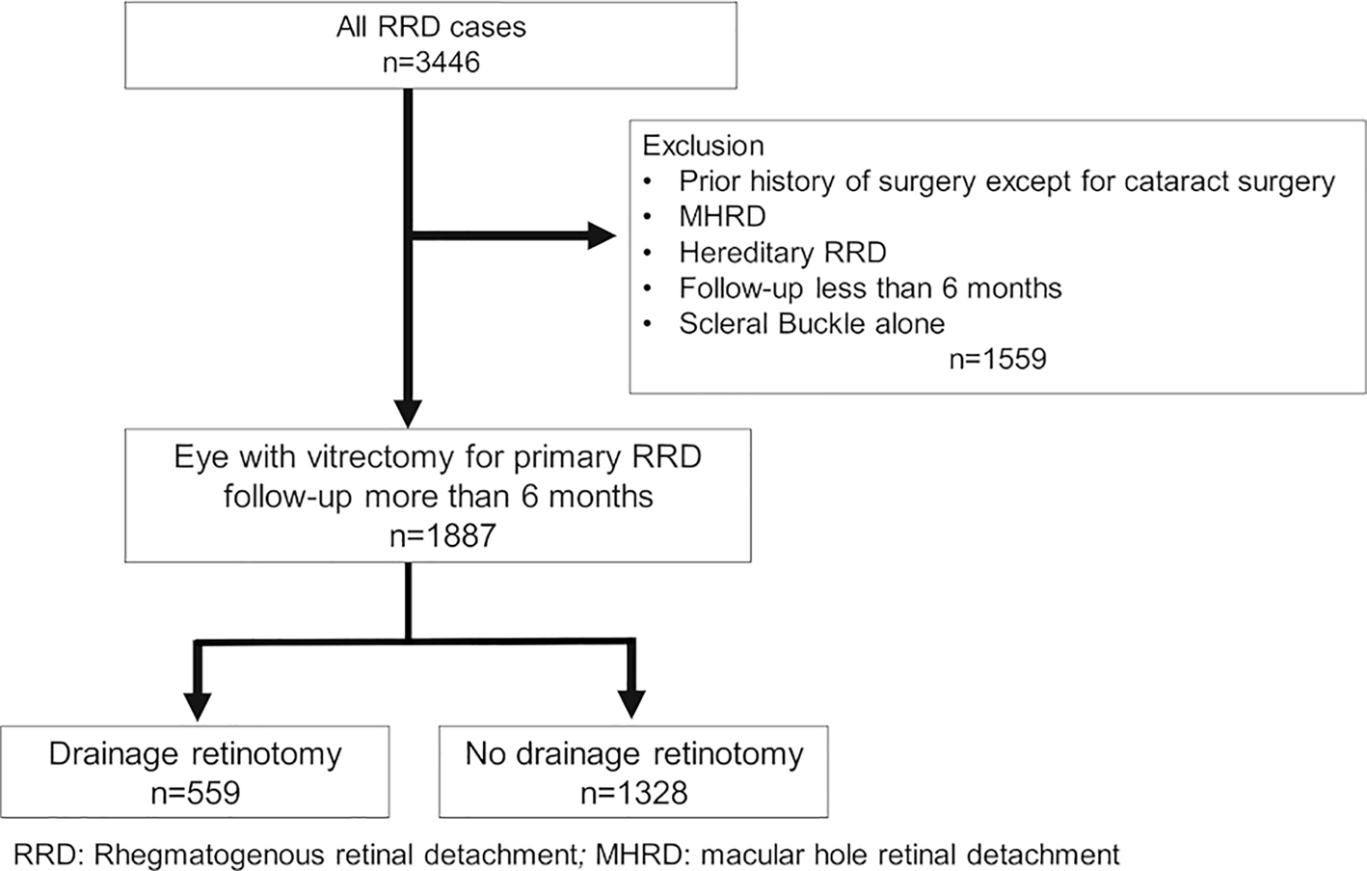
Flow chart of the present retrospective study. *RRD* rhegmatogenous retinal detachment, *MHRD* macular hole retinal detachment.

Figure 1 shows a flow chart of the patient selection process. Of 3446 eyes, 1887 met the inclusion criteria. Of these 1887 eyes, 559 underwent vitrectomy with drainage retinotomy and 1328 underwent vitrectomy without drainage retinotomy.

Table 1 shows the demographic characteristics of eyes with or without drainage retinotomy. We observed several significant differences between these two groups of eyes. The drainage retinotomy group exhibited older age (60.2 vs. 59.0 years, p = 0.007) and lower intraocular pressure (12.3 vs. 13.3 mmHg, p < 0.001). The drainage retinotomy group also had larger RD areas (2.3 vs. 2.0 quadrants, p < 0.001), a higher incidence of choroidal detachment (6.6% vs. 3.2%, p = 0.002). Additionally, the drainage retinotomy group had higher percentages of pseudophakic eyes (22.9% vs. 17.9%) and macula off RRD (58.1% vs. 46.1%)., and more severe PVR grades (grade B or C; 16.5% vs. 7.8%). Internal limiting membrane peeling was more frequently performed in the drainage retinotomy group (26.3% vs. 21.9%, p = 0.042), the surgical time was longer (92.4 vs. 80.0 min, p < 0.001) than in the no drainage retinotomy group. Moreover, the drainage retinotomy group had a higher frequency of silicone oil tamponade usage (11.0% vs. 5.4%).

**Table 1:** Clinical characteristics and surgical details.

	Drainage retinotomy (n = 559)	No drainage retinotomy (n = 1328)	P
Age, years	60.2 ± 13.3	59.0 ± 11.5	0.007
Sex, male	386 (69.1)	924 (69.6)	0.83
Eye, right	225 (53.8)	217 (51.9)	0.63
Axial length, mm	(n = 460) 25.34 ± 1.77	(n = 1052) 25.39 ± 1.82	0.50
Baseline logMAR BCVA	(n = 553) 0.678 ± 0.804	(n = 1324) 0.604 ± 0.828	0.075
Intraocular pressure, mmHg	(n = 555) 12.3 ± 3.9	(n = 1316) 13.3 ± 3.5	< 0.001
Lens status			
Aphakia	4 (0.7)	14 (1.1)	0.043
Phakia	427 (76.4)	1075 (81.0)	
Pseudophakia	128 (22.9)	239 (17.9)	
Macular status			
On	324 (58.0)	234 (41.9)	< 0.001
Off	234 (41.9)	709 (53.4)	
Unknown	1 (0.2)	13 (1.0)	
RD area, quadrants	2.3 ± 0.9	2.0 ± 0.9	< 0.001
Choroidal detachment	37 (6.6)	43 (3.2)	0.002
Maximum retinal break location			
Upper temporal	267 (47.8)	755 (56.9)	0.004
Upper nasal	144 (25.8)	276 (20.8)	
Lower temporal	98 (17.5)	203 (15.3)	
Lower nasal	48 (8.6)	84 (6.3)	
Posterior pole	2 (0.4)	10 (0.8)	
Retinal breaks	(n = 536)	(n = 1310)	0.74
1	267 (49.8)	685 (52.3)	
2	133 (24.8)	297 (22.7)	
3	62 (11.6)	148 (11.3)	
≥ 4	74 (13.8)	180 (13.7)	
PVR grade			< 0.001
N	467 (83.5)	1225 (92.2)	
B	43 (7.7)	60 (4.5)	
C	49 (8.8)	43 (3.2)	
Combination of scleral buckling	41 (7.3)	102 (7.7)	0.84
Combination of cataract surgery	161 (28.8)	430 (32.4)	0.13
ILM peeling	147 (26.3)	291 (21.9)	0.042
Surgical time	92.4 ± 43.2	80.0 ± 37.7	< 0.001
Tamponade	(n = 557)	(n = 1272)	< 0.001
Air	26 (4.7)	243 (19.1)	
SF_6_	457 (82.1)	928 (73.0)	
C_3_F_8_	13 (2.3)	32 (2.5)	
SO	61 (11.0)	69 (5.4)	

Data are presented as mean ± standard deviation or n (%).

*BCVA* best-corrected visual acuity, *RD* retinal detachment, *PVR* proliferative vitreoretinopathy, *ILM* inner limiting membrane, *SF*_*6*_ sulfur hexafluoride, *C*_*3*_*F*_*8*_ octafluoropropane, *SO* silicone oil.

### Primary anatomical success rate and visual outcomes

In the drainage retinotomy group, secondary surgeries were performed within 6 months in 81 eyes (29 [5.2%] for silicone oil removal, 8 [1.4%] for ERM, 35 [6.3%] for recurrent RRD, and 9 [1.6%] for other conditions). In the no drainage retinotomy group, secondary surgeries were performed in 116 eyes (32 [2.4%] for silicone oil removal, 14 [1.8%] for ERM, 60 [4.5%] for recurrent RRD, and 10 [0.8%] for other conditions). The primary anatomical success rate was 95.0% across all cases. The primary anatomical success rate was not significantly different between the groups with and without drainage vitrectomy (6.3% vs. 4.5%, P = 0.13). Likewise, the rate of secondary vitrectomy surgery for ERM within 6 months after the initial surgery was not significantly different between the two groups (1.4% vs. 1.1%, P = 0.49). The mean Log MAR BCVA at 6 months after vitrectomy was 0.20 in eyes with drainage retinotomy and 0.11 in eyes without drainage retinotomy, showing a significant difference (P < 0.001).

To ensure balance in the patients’ background characteristics and surgical procedures, we created a propensity score-matched dataset consisting of two pairs of eyes with and without drainage retinotomy. Table 2 displays the demographic characteristics of the study population with and without drainage retinotomy in the score-matched dataset. We found no significant differences in the matched baseline variables between the groups in the propensity score-matched data.

**Table 2:** Propensity score-matched clinical characteristics, surgical details, and surgical outcomes.

	Drainage retinotomy (n = 544)	No drainage retinotomy (n = 544)	P
Age, years	59.8 ± 12.9	59.4 ± 12.4	0.53
Sex, male	375 (68.9)	372 (68.4)	0.90
Eye, right	298 (54.8)	283 (52.0)	0.39
Axial length, mm	(n = 449) 25.37 ± 1.77	(n = 406) 25.18 ± 1.88	0.10
Baseline logMAR BCVA	0.668 ± 0.798	0.681 ± 0.825	0.99
Intraocular pressure, mmHg	12.6 ± 3.6	12.8 ± 3.6	0.13
Lens status			
Aphakia	2 (0.5)	3 (0.7)	0.70
Phakia	362 (82.7)	369 (84.3)	
Pseudophakia	74 (16.9)	66 (15.1)	
Macular status			
On	233 (42.8)	234 (43.0)	1.0
Off	311 (57.2)	310 (57.0)	
RD area, quadrants	2.2 ± 0.9	2.2 ± 0.9	0.65
Choroidal detachment	34 (6.3)	23 (4.2)	0.17
Maximum retinal break location			
Upper temporal	263 (48.4)	253 (46.5)	0.71
Upper nasal	139 (25.6)	145 (26.7)	
Lower temporal	94 (17.3)	105 (19.3)	
Lower nasal	46 (8.5)	38 (7.0)	
Posterior pole	2 (0.4)	3 (0.6)	
Retinal breaks			
1	258 (47.4)	275 (50.6)	0.70
2	130 (23.9)	119 (21.9)	
3	62 (11.4)	64 (11.8)	
≥ 4	94 (17.3)	86 (15.8)	
PVR grade			
N	463 (85.1)	460 (84.6)	0.67
B	37 (6.8)	44 (8.1)	
C	44 (8.1)	40 (7.4)	
Combination of scleral buckling	35 (6.4)	41 (7.5)	0.55
Combination of cataract surgery	155 (28.5)	149 (27.4)	0.74
ILM peeling	101 (24.2)	101 (24.2)	1.0
Surgical time	91.3 ± 41.6	92.3 ± 40.5	0.48
Tamponade			
Air	26 (4.8)	22 (4.0)	0.72
SF_6_	454 (83.5)	449 (82.5)	
C_3_F_8_	13 (2.4)	18 (3.3)	
SO	51 (9.4)	55 (10.1)	
Six-month postoperative BCVA	0.181 ± 0.387	0.166 ± 0.413	0.23
Primary anatomical success rate	510 (93.8)	520 (95.6)	0.22
Secondary surgery for ERM	8 (1.5)	7 (1.3)	1.0

Data are presented as mean ± standard deviation or n (%).

*BCVA* best-corrected visual acuity, *RD* retinal detachment, *PVR* proliferative vitreoretinopathy, *ILM* inner limiting membrane, *SF6* sulfur hexafluoride, *C3F8* octafluoropropane, *SO* silicone oil, *ERM* epiretinal membrane.

In the propensity score-matched dataset, there was no significant difference in BCVA between the two groups at 6 months after vitrectomy (Log MAR: 0.181 vs. 0.166, P = 0.23). In the drainage retinotomy group, secondary surgeries were performed within 6 months in 75 eyes (26 for silicone oil removal, 8 for ERM, 34 for RRD, and 7 for other conditions). In the no drainage retinotomy group, secondary surgeries were performed in 75 eyes (28 for silicone oil removal, 7 for ERM, 24 for RRD, and 6 for other conditions). There was no significant difference between the two groups in the primary anatomical success rate (6.3% vs. 4.4%, P = 0.22) or the rate of secondary surgery for ERM (1.5% vs. 1.3%, P = 1.0).

## Discussion

We assessed the influence of drainage retinotomy on the outcomes of RRD surgery through analysis of the J-RD Registry. We found that drainage retinectomy tended to be used in more complicated cases, including those involving macula-off RD, larger RD areas, choroidal detachment, and higher PVR grades. Notably, the success rate remained unaffected in the drainage retinotomy group. Additionally, we found no significant impact of drainage retinotomy on postoperative BCVA and the incidence of secondary operations for ERM within 6 months after drainage retinectomy. These results suggest the efficacy of drainage retinectomy as a valuable surgical procedure for RRD repair.

In the present study using a nationwide registry in Japan, drainage retinotomy was performed in approximately 30% of cases. The drainage retinotomy group exhibited a higher percentage of macula-off RRD, larger RD areas, a higher incidence of choroidal detachment, more severe PVR grades, and longer surgical times than the no drainage retinotomy group. These data imply that drainage retinotomy was performed in more complicated cases of RD. Although drainage of subretinal fluid is an important procedure in RD surgery, recent reports have proposed that complete intraoperative subretinal fluid drainage may not be imperative for vitrectomy in RD^16^. Nonetheless, residual subretinal fluid can become trapped and mechanically displaced, which might be caused by conditions such as retinal folds^17^. retinal displacement^18^, and an insufficient tamponade effect for inferior breaks^19^. Our data show the current trends in the practice of retina surgeons, indicating that drainage retinectomy is more likely to be performed in complicated cases.

Drainage retinotomy had no impact on the primary anatomical success rate in this study. The influence of drainage retinectomy on the primary success rate remains a subject of debate. Ohara et al.^7^ recently identified drainage retinotomy as a risk factor for surgical failure after PPV in patients with uncomplicated RRD. They proposed that avoiding drainage retinotomy could enhance surgical success. However, their study excluded complicated cases such as those involving PVR grade C1 or worse, giant retinal tears (≥ 90°), or traumatic RD and did not indicate whether perfluorocarbon liquid was used in the surgical procedure. Our study included these complicated cases of RRD. Moreover, our study used data from the J-RD Registry, thus reflecting real-world cases with a diverse range of complications and involving ophthalmological surgeons with varying levels of experience. However, other recent studies have explored different drainage techniques. McKay et al.^10^ compared three methods and found no significant difference in the primary anatomical success rate among cases involving peripheral retinal breaks, posterior retinotomy, or the use of perfluorocarbon liquid. Furthermore, in their prospective study, Kumari et al.^9^ reported a similar anatomical success rate between posterior retinotomy and perfluorocarbon liquid-assisted drainage. Similar to these reports, our results suggest that drainage retinectomy is not significantly linked to surgical failure.

Some recent retrospective reports have suggested that drainage retinotomy is a risk factor for the development of an ERM^7,20^. Previously, Ishikawa et al. reported that drainage retinectomy are more likely to develop ERM after vitrectomy using J-RD registry data. In our study, however, secondary surgery for an ERM was performed within 6 months in 8 (1.4%) eyes with drainage retinotomy and 14 (1.1%) eyes without drainage retinotomy, and the difference was not statistically significant. This difference could come from the evaluation of ERM. The previous study evaluated the incidence of ERM development. Compared to the previous J-RD study, our data focused on the secondary surgery for ERM not for the presence of ERM. The occurrence of ERM development following PPV for RRD appears to be relatively common. A recent study indicated that half of the eyes treated by PPV for primary RRD had visible ERMs on optical coherence tomography. However, visual acuity was not impacted in the majority of these eyes, and only 5% of eyes required subsequent surgery for ERM removal^21^. Therefor, to evaluate short-term outcomes, our data evaluated the re-surgery rate and there was no significant difference between the two groups. Moreover, there was no significant difference in BCVA at 6 months after treatment between the two groups. In our dataset, the rate of vitrectomy for an ERM within 6 months was 1.2% (22 eyes), which is a lower rate than in previous studies. With a more extended follow-up period, we may identify ERMs that require surgical intervention. However, drainage retinectomy did not affect the short-term postoperative outcomes in this study.

The present study had several limitations. First, because of its retrospective design, we could not exclude the possibility of unidentified confounders. In this registry, the surgical procedure and data collection were not standardized. To reduce confounders, we performed a propensity score-matching analysis; however, the inherent limitations of retrospective studies persist. Second, the observation period was short. The 6-month endpoint may not have fully captured the long-term visual prognosis and late-onset complications. Finally, the registry data used in this study did not specify the location of drainage retinotomy, adding ambiguity to the analysis.

In conclusion, our findings suggest that drainage retinectomy, which is likely used for more complicated cases, does not increase the risk of surgical failure or a decrease in postoperative BCVA in the short term. The results demonstrate the effectiveness of drainage retinectomy as an option for intraoperative drainage of subretinal fluid in RD surgery.

## Methods

The J-RD Registry, maintained by the Japanese Retina and Vitreous Society, is a private database. It encompasses records of retinal detachment (RD) surgeries performed from February 2016 to March 2017. For each surgery, the registry contains comprehensive patient details along with in-depth data regarding the underlying cause and nature of RD, the surgical approach employed, and the resulting surgical outcomes. Essential among the surgical outcome data are postoperative visual acuity measurements at 1, 3, and 6 months as well as information pertaining to retinal restoration.

The Institutional Review Board of Kagoshima University approved the study (approval no. 140093), and the study was conducted in accordance with the guidelines outlined in the Declaration of Helsinki. The review board waived the requirement for informed consent because of the deidentification of all information within the J-RD Registry database.

### Patients and exclusion criteria

The number of cases in the database was 3446, and the details of the patients’ demographics have been published^11^. In this study, we excluded eyes that underwent scleral buckling and those that were followed up for < 6 months. We also excluded eyes with a history of surgery other than cataract surgery, with macular hole RD, and with hereditary RRD (Fig. 1).

### Data collection

We collected the following baseline characteristics: sex, age at the time of surgery, best-corrected visual acuity (BCVA) measured using the logarithm of the minimum angle of resolution scale, intraocular pressure, axial length, lens status, maximum retinal break location, number of retinal breaks, quadrants of RD area, macular status (retinal detachment involved fovea), and proliferative vitreoretinopathy (PVR) grade. Surgical data included the performance of scleral buckling, the performance of cataract surgery, the operation time, the performance of internal limiting membrane peeling, the performance of drainage retinotomy, and any tamponade agents used. Postoperative clinical data included additional surgeries required for re-detachment during the first 6 months postoperatively and the BCVA at 1, 3, and 6 months postoperatively.

### Study endpoints

The primary endpoint was the comparison of postoperative BCVA at 6 months between eyes with and without drainage retinectomy. The secondary endpoints were comparisons of the primary anatomical success rate, the rate of secondary surgery for epiretinal membrane (ERM), and the baseline characteristics between the two groups.

### Statistical analysis

The baseline characteristics and surgical procedures were compared between the two groups using the χ^2^ test or Fisher’s exact test for categorical variables and the t test (parametric data) or Mann–Whitney *U* test (nonparametric data) for continuous variables. We conducted propensity score matching to evaluate the risk of requiring drainage retinotomy. We used multiple logistic regression models to identify the clinical parameters necessary to produce a propensity score. For each group, we performed propensity score matching using the resulting dataset; 1:1 paired matching was used to eliminate sample size bias, and nearest-neighbor matching with a caliper of 0.1 was used to exclude pairs with disparate propensity scores. After propensity score matching, we compared the surgical outcomes between the two groups using the Wilcoxon–Mann–Whitney test. JMP software version 15.2.0 (SAS Inc., Cary, NC, USA) was used for all statistical analyses, and a P value of < 0.05 was considered statistically significant.

## Data Availability

The datasets used and/or analyzed during the current study are available from the corresponding author upon reasonable request.
